# Clinician's attitude to enteral nutrition with percutaneous endoscopic gastrostomy: a survey in China

**DOI:** 10.1186/s41043-021-00264-9

**Published:** 2021-09-26

**Authors:** Yijie Zhang, Chen Ma, Chenxi Li, Qian Chen, Meifen Shen, Yuyu Wang

**Affiliations:** 1 Department of Neurosurgery, The First Affiliated Hospital of Soochow University, No.188 Shizi Street, Suzhou, China; 2 School of Nursing, Medical College of Soochow University, No.188 Shizi St, Suzhou, China

**Keywords:** Percutaneous endoscopic gastrostomy (PEG), Enteral nutrition, Decision making, Attitude, Chinese doctors

## Abstract

**Background:**

Percutaneous endoscopic gastrostomy (PEG) is recommended for long-term enteral nutrition. However, long-term nasogastric (NGT) feeding is still commonplace in China. We surveyed Chinese clinicians’ opinions toward PEG feeding in order to identify the potential barriers to acceptancy of PEG feeding.

**Methods:**

A self-reported questionnaire was developed and distributed to 600 doctors. Five-point Likert scales were used for most responses.

**Results:**

Of 525 respondents, the mainly nutritional support method was NGT while PEG was less used. Doctors working in the tertiary class A hospitals and radiotherapy department were more likely to choose PEG feeding (p = 0.000). Overall, 241 (46%) participants did not know PEG and 284 (54%) have different understanding degree of PEG. Age (p = 0.002), working life (p = 0.044) and professionalism (p = 0.005) were significantly related to the understanding of PEG. Levels of agreement was high (score of 3.47) for using PEG in patients with prolonged stroke-associated dysphagia. There was high agreement level in the statement that PEG was unnecessary when NGT could sustain the basic needs of patients, though better outcome can be predicted with PEG feeding. The highest scoring factor (score of 3.91) that influenced clinicians’ choice of PEG was resistance from patients and families and the second one was the poor cooperation among departments (score of 3.80).

**Conclusions:**

Doctors’ insufficient knowledge of PEG feeding, resistance from patients and families, poor cooperation among departments, all these factors leading physicians to prefer more conservative treatment to avoid disputes rather than better ones.

**Supplementary Information:**

The online version contains supplementary material available at 10.1186/s41043-021-00264-9.

## Background

Patients who suffer from prolonged dysphagia associated with head and neck cancers, stroke, and chronic neurodegenerative conditions usually require long-term enteral nutritional support with percutaneous endoscopic gastrostomy feeding tubes (PEG) instead of nasogastric feeding tubes (NGT) [1]. Some studies advocated for NGT before the establishment of PEG tube feeding as it is a safer option with less risk of infection or granulation tissue formation associated with PEG tube placement [2–4]. Nevertheless, PEG feeding has been demonstrated in studies to have several advantages over long-term NG tube feeding, including a lower rate of complications [4, 5], better nutritional status [6, 7] and a higher survival rate [8]. In patients on long-term feeding, the risk of pneumonia was reported to be significantly higher in patients with NGT than in those with PEG (adjusted hazard ratio = 2.85) [9].

However, the acceptance of PEG tube feeding seems to vary considerably among different countries. There is a clear contrast between Asian and Western about the attitudes toward PEG feeding of clinicians as well as the clinical practice [10–12]. In North America, PEG tubes are most frequently recommended for long-term enteral nutrition by HCPs with nearly 40% of them holding the belief that PEG feeding is the standard of care for patients [13]. Similarly, the use of PEG feeding tube in long-term care and community settings is now common place in European countries. Nevertheless, a survey conducted amongst residential elderly care institutions in Taiwan, 80% of patients with dysphagia were found to be on long-term NG feeding [11]. In a research from Malaysia, the majority of elderly patients with dysphagia in residential care were found to be on long-term NG feeding, despite having inadequate calorie intake and significant malnutrition [12].

Numerous studies have evaluated the potential difficulties may be encountered in the practice of long-term enteral feeding with PEG tubes. Nevertheless, there are few reports about Chinese doctors' attitudes towards PEG feeding except a research published in 2018 which surveyed the opinion of radiation oncologists on PEG feeding [14]. In order to identify the potential barriers to the acceptance and delivery of PEG tube feeding among clinicians in China, a survey was conducted with a questionnaire includes 11 questions about PEG decision-making process to analyze the clinicians' attitude to enteral nutrition with PEG.

## Methods

An observational study was conducted to investigate the attitudes of clinicians in Jiangsu Province towards the use of PEG for enteral nutrition. Participants for this study were selected through a convenience sampling method. This study was approved the Ethics Committee of Soochow University.

### Questionnaire development

In an interdisciplinary meeting, a general surgeon who places PEG tubes, a rehabilitation department physician, a speech language pathologist (SLP), a neurology registered nurse, and a neurosurgery registered nurse derived consensus on optimal decision-making practices for PEG placement. Based on published literature and experience, the work team created a questionnaire which was used in Department of Neurosurgery of the First Affiliated Hospital of Soochow University for a pilot survey and made some revisions. The Cranbach α of the final questionnaire was 0.828, the Kaiser–Meyer–Olkin test of sampling showed to be adequate (KMO = 0.849) and the Bartlett test of sphericity was significant (p < 0.001), suggesting that the questionnaire has good reliability and construct validity.

### Survey

Approval or written consent is not necessary because participation in the survey is on an entirely voluntary basis and consent is given by clicking the "I agree" box before answering the survey questions. Participants can withdraw at any time, responses were kept confidential and data were reported only in the aggregate.

The questionnaire surveyed the current methods of enteral nutrition mainly used in different departments, physicians’ understanding of PEG, the decision-making process of PEG, and the reasons for unwilling to use PEG (Additional file 1: Table s1). Answer options were based on 5-point Likert scales. Surveys were emailed to 600 clinicians, including general surgery, neurology, neurosurgery, rehabilitation department, general surgery, gastroenterology, oncology, radiotherapy department and others (obstetrics & gynaecology, endocrinology, emergency care, rheumatology, paediatrics, urology, respiratory medicine). One to two days prior to the survey distribution, messages were emailed to the physicians in order to inform them that an upcoming survey would take them about 5 min to complete.

**Table 1:** Demographic characteristics of the participants (n = 525)

Characteristic	Participants
Total, n	525
Departments, n (%)	
ICU	13 (2.5)
Neurology	45 (8.6)
Neurosurgery	27 (5.1)
Rehabilitation Dept	40 (7.6)
General surgery	33 (6.3)
Gastroenterology	34 (6.5)
Oncology	29 (5.5)
Radiotherapy Dept	20 (3.8)
Other	284 (54.1)
Age, years, n (%)	
20–30 years	67 (12.8)
30–40 years	322 (61.3)
40–50 years	96 (18.3)
> 50 years	40 (7.6)
Educational background, n (%)	
College	10 (1.9)
Bachelor	290 (55.2)
Master	181 (34.5)
Doctor	44 (8.4)
Degree of the hospital, n (%)	
Tertiary class A hospital	134 (25.5)
Tertiary class B hospital	386 (73.5)
Secondary class A hospital	2 (0.0)
Secondary class B hospital	3 (0.0)
Working life, n (%)	
< 5 years	70 (15.2)
≥ 5 years	445 (84.8)
Professional level, n (%)	
Resident	116 (22.1)
Attending	241 (45.9)
Deputy Chief	113 (21.5)
Chief	55 (10.5)
Degree of understanding of PEG, n (%)	
Know nothing	124 (23.5)
Do not know much	117 (22.3)
Know some of it	193 (36.8)
Know well	55 (10.5)
Very clear	36 (6.9)

### Definitions

Participants were divided into “ < 40 years” and “ ≥ 40 years” based on age and “ < 5 years” and “ ≥ 5 years” based on years of working life. The professionalism of clinicians was classified as “Resident” “Attending” “Deputy Chief” and “Chief”. The degree of understanding of PEG has been categorized into five levels: “Known nothing” means the clinicians almost never heard of PEG; “Do not know much” means that the doctor has heard of PEG but does not know much more about it; “Know some of it” means that the doctor has some knowledge of PEG, such as how to perform PEG and the relevant indications; “Know well” means that the doctor means that the doctor is familiar with PEG, understands its indications and contraindications, and can make rational decisions based on the patient's clinical needs; “Very clear” means that the doctor is fully knowledgeable about PEG, can perform the operation independently and make appropriate nutritional decisions, and can manage complications related to tube placement.

### Analysis

“Level of agreement” scores were reported as means and standard deviations of aggregated data based on the answer frequencies for the 5-point Likert scales (Some items were reverse coded). Chi-squared test was used to assess associations between demographic variables and understanding degree of clinicians towards PEG. Mann–Whitney U test or Kruskal–Wallis test was used for categorical variables. For post hoc analysis of differences between the 9 departments, and between different professional status of physicians, data were analyzed by one-way analysis of variance. Statistical analysis was performed by two investigators using SPSS software (version 25.0 for Windows VR (IBM SPSS, Armonk, NY: IBM Corp, USA). A two-sided analysis was used and p < 0.05 was regarded as statistically significant.

## Results

Of the 600 questionnaires issued, 525 forms were completed correctly and the completion rates is 87.5%. 501 forms from Jiangsu Province and the others from Shanghai, Anhui, Hubei, Shanxi, Sichuan, Tianjin, Xinjiang, Zhejiang and Chongqing provinces. The respondents are more concentrated in the age between 30–40 years old (322/61.3%) and most of them work in the tertiary class A and class B hospitals (520/99.0%). 116 participants are resident, 241 are attending, 113 are deputy chief and 55 are chief. Most doctors are bachelors (290/55.2%) and masters (181/34.5%). The details of demographic data are shown in Table 1.

### Frequency of use of different enteral nutrition methods in different departments

**Fig. 1:**
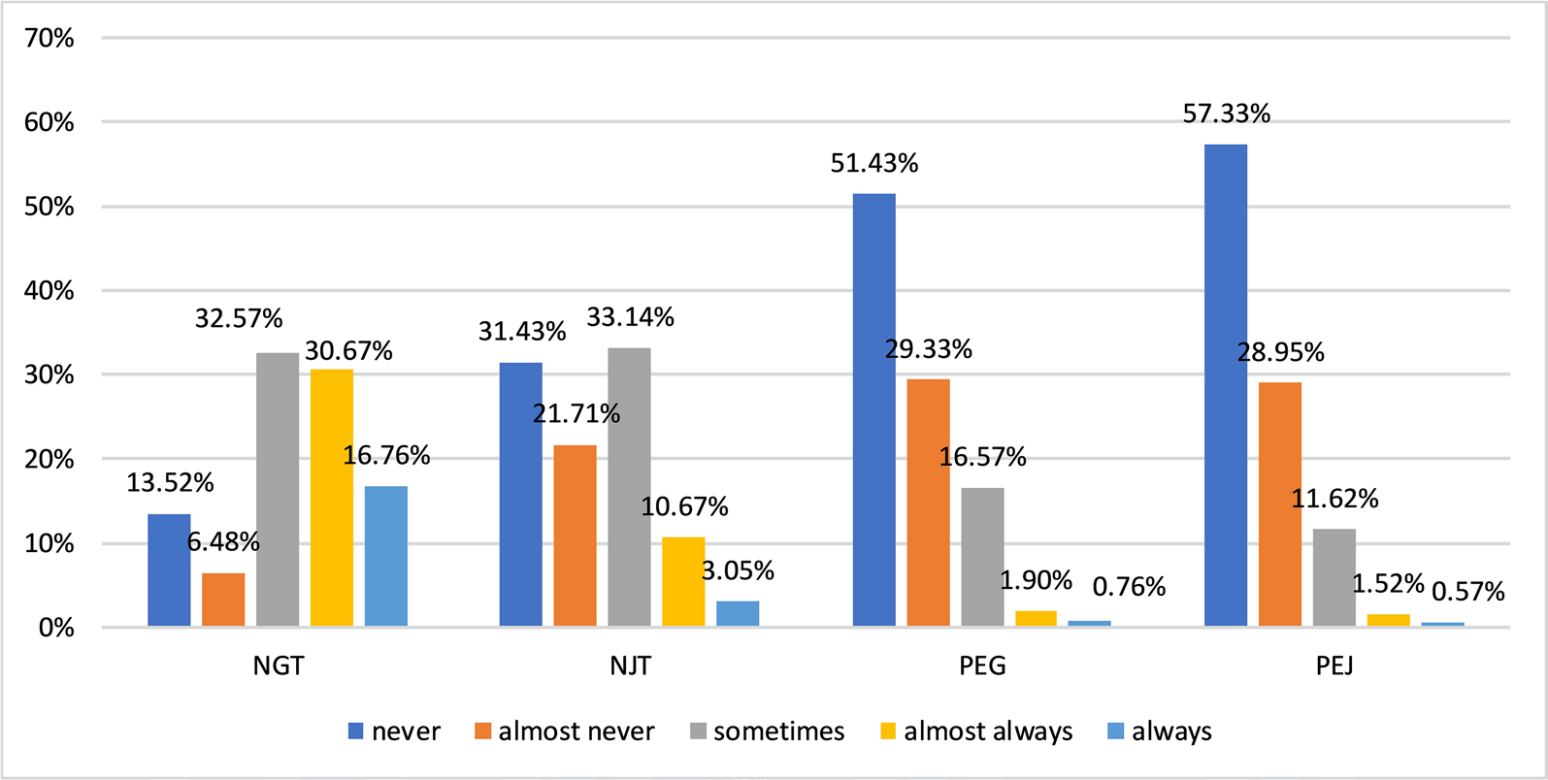
Frequency of use of different enteral nutritional methods

Clinicians were asked to choose the frequency of use of different enteral nutritional methods including nasogastric tube (NGT), nasal-intestinal tube (NJT), percutaneous endoscopic gastrostomy (PEG) and percutaneous endoscopic jejunostomy (PEJ) according to their clinical reality. The options indicated frequency were range from “*never*”, “*almost never*” or “*sometimes*” to “*almost always*” or “*always*”. There was significant difference (p = 0.000) among the frequency of use of different nutritional methods and the result showed that NGT was more commonly used for nutritional support (Fig. 1).

**Fig. 2:**
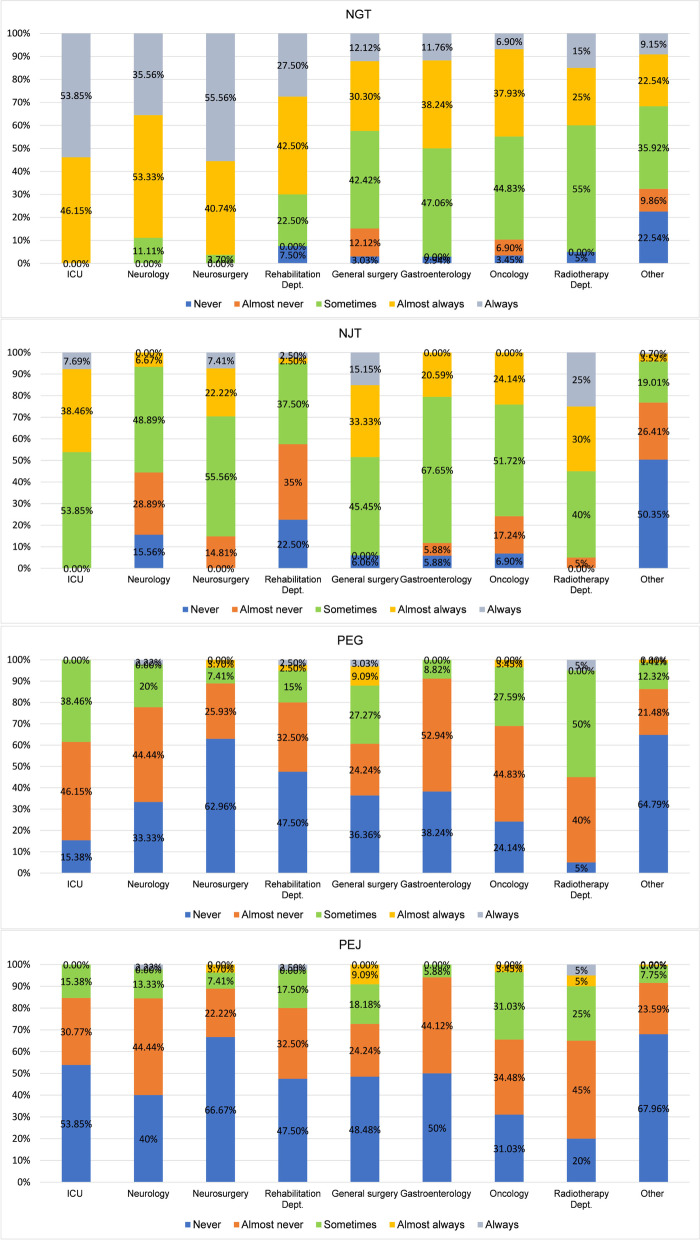
Nutritional decisions of different departments

Additionally, we found that nutritional decisions varied among departments (Fig. 2). Neurosurgery chose NGT as its optimal options (55.6% “*always*” choice) more often than other departments. NJT was the secondary choice which was widely accepted in ICU, neurosurgery, general surgery, gastroenterology, oncology and radiotherapy departments. Nevertheless, when it came to PEG and PEJ, the most commonly selected options were “*never*” or “*almost never*”. There was significant difference (p = 0.000) among departments in frequency of choosing PEG for nutritional support. The largest proportion of the participants (55%) of the radiotherapy department reported using PEG for nutritional support.

**Fig. 3:**
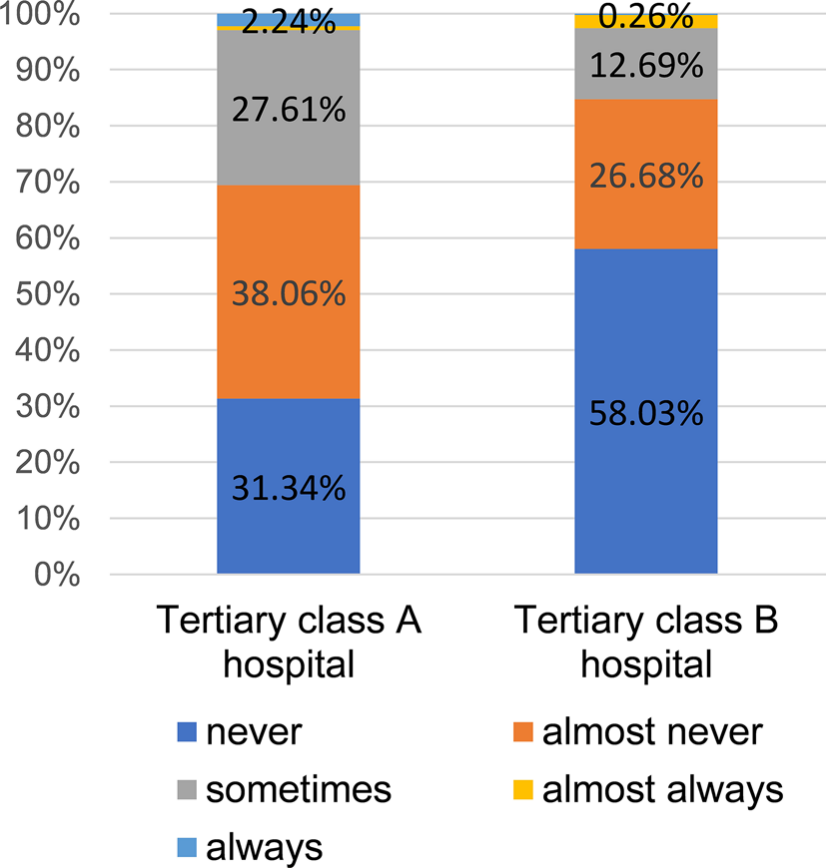
Frequency of PEG using in different level hospital

The nutritional decision-making also varied between different levels’ hospitals (Fig. 3). Since the vast majority of doctors participating in the survey came from the tertiary class A and class B hospitals (134 and 386, respectively), our analysis only included these two levels. The frequency of PEG use was varied in different levels of hospitals (p = 0.000). The high-grade hospitals (“*sometimes*” & “*almost always*” & “*always*” accounted for 30.6%) were more likely to choose PEG as a long-term nutritional method for patients than the lower ones (“*sometimes*” & “*almost always*” & “*always*” accounted for 15.3%).

### The understanding of PEG

We also investigated the 525 clinicians’ knowledge about PEG, 124 (23.5%) knew nothing, 117 (22.3%) didn’t know much, 193 (36.8%) knew some, 55 (10.5%) knew well and only 36 (6.9%) participants have a good understanding about PEG. The degree of the understanding about PEG in relation to their demographic characteristics is shown in Table 2. Age (p = 0.002), working life (p = 0.044) and professionalism (p = 0.005) were significantly related to the understanding of PEG. For example, clinicians with work experience ≥ 5 years, age ≥ 40 years old and a higher professional status had a better understanding of PEG. Furthermore, clinicians with a doctorate (72.7%, p = 0.006) degree knew more than that with other degrees (51.0%). Hospital classification and different departments also had an impact on doctors’ understanding of PEG. Doctors working in higher-level hospitals (72.4%, p < 0.001) performed better than doctors working in secondary hospitals (47.9%) and that whose working departments were ICU (92.3%), general surgery (90.9%), oncology (82.4%) and radiotherapy (80.0%) had a deeper understanding about PEG (p < 0.001).

**Table 2:** Demographic characteristics and clinicians’ understanding of PEG (n = 525)

Characteristic	Know PEG (n = 284)	Do not know PEG (n = 241)	p
Age			
< 40 years old	195 (50.1)	194 (49.9)	0.002
≥ 40 years old	89 (31.3)	47 (19.5)	
Departments			
ICU	12 (92.3)	1 (7.7)	< 0.001
Neurology	27 (60.0)	18 (40.0)	
Neurosurgery	14 (51.9)	13 (48.1)	
Rehabilitation Dep	21 (52.5)	19 (47.5)	
General surgery	30 (90.9)	3 (9.1)	
Gastroenterology	28 (18.4)	6 (15.6)	
Oncology	24 (82.4)	5 (17.2)	
Radiotherapy Dept	16 (80.0)	4 (20.0)	
Other	112 (39.4)	172 (60.6)	
Educational background			
College	2 (20.0)	8 (80.0)	0.006
Bachelor	148 (51.0)	142 (49.0)	
Master	102 (56.4)	79 (43.6)	
Doctor	32 (72.7)	12 (27.3)	
Degree of the hospital			
Tertiary class A hospital	97 (72.4)	37 (61.3)	< 0.001
Tertiary class B hospital	185 (47.9)	201 (52.1)	
Working life			
< 5 years	35 (43.8)	45 (56.3)	0.044
≥ 5 years	249 (56.0)	196 (44.0)	
Professional level			
Resident	51 (44.0)	65 (56.0)	0.005
Attending	126 (52.3)	115 (47.7)	
Deputy Chief	68 (60.2)	45 (39.8)	
Chief	39 (70.9)	16 (29.1)	

### Insufficient knowledge of the indications and contraindications for PEG

For questions 9–11, we only investigated participants who chose the options included “*know some about it*”, “*know well*” and “*very clear*” in the question about the knowledge of PEG and 284 clinicians were included at last. Table 3 emphasizes the very low scores with the statements that PEG feeding tubes are contraindicated in advanced dementia and at end of life (These two items were reverse coded items: 1 = *Always*, 2 = *Almost always*, 3 = *Sometimes*, 4 = *Almost always*, 5 = *Never*). Surprisingly, there is a high degree of consistency in the choice of nutritional support for patients with long-term dysphagia after stroke, with a score of 3.47. There were no significant differences in responses between departments, nor among different professional level of clinicians.

**Table 3:** Level of agreement to the use of PEG in different contexts (n = 284)

Would you recommend PEG for the following patients who may need long-term nutritional support	Level of agreement Mean ± SD (scale 1–5)
Patients with advanced dementia	2.92 ± 1.05
Patients in terminal or palliative care	2.81 ± 1.03
Patients with multiple sclerosis or amyotrophic lateral sclerosis	3.34 ± 1.06
Patients with maxillofacial tumor	3.43 ± 1.01
For stroke patients, I will place NGT/ NJT after the diagnosis of oropharyngeal swallowing disorder. If the swallowing disorder persists for 2 weeks, I will ask the patient's wishes, consider and recommend using PEG feeding	3.47 ± 1.00
Patients with intolerance of nasogastric tube and nasal jejunal tube with complications such as reflux, gastroparesis, and gastric retention	3.56 ± 1.00

[1 = *Never*, 2 = *Almost never*, 3 = *Sometimes*, 4 = *Almost always*, 5 = *Always*]

*1&2 item use reverse scoring: 1 = *Always*, 2 = *Almost always*, 3 = *Sometimes*, 4 = *Almost always*, 5 = *Never*

### High degree of consistency in the decision-making process for PEG feeding tubes

For the statement about the decision-making process for PEG, the score was very high except the second item, the scores were ranging from 4.13–4.24. Table 4 lists the discussion topics and average levels of agreement for each. Clinicians reached a high consensus on the protection of patients’ rights and interests for decision-making. Nevertheless, for the statement that PEG was not necessary when NGT could sustain the basic needs of patients, though better outcome can predict with PEG feeding, clinicians’ score was very low (this item was reverse coded), almost half of the participants (49.9%) chose “*Somewhat agree*” and 68 (12.95%) chose “*Completely agree*”. There were no significant differences in responses among departments.

**Table 4:** Level of agreement with use of discussion topics in decision making (n = 284)

Do you agree with the following statement about PEG?	Level of agreement Mean ± SD (scale 1–5)
PEG can only be used in accordance with the patient's condition and his own wishes	4.20 ± 0.73
Although patients with PEG feeding may have a better prognosis, NGT/ NJT can already sustain the basic needs of patients, there is no need for PEG	2.40 ± 0.90
I will recommend the type of enteral nutrition to be used according to the patient's condition and guidelines. If it is contrary to the patient's wishes, I will communicate with them to explain the reasons for making decisions. In case of conflict, the patient's wishes shall prevail	4.14 ± 0.67
If the PEG placement process can be simplified, it will be more conducive for me to make PEG decisions	4.13 ± 0.65
Establishing a multi-disciplinary nutrition decision-making team will help me better choose nutrition support methods	4.24 ± 0.69

[1 = *Disagree*, 2 = *Somewhat disagree*, 3 = *Neither agree nor disagree*, 4 = *Somewhat agree*, 5 = *Completely agree*]

*Item 2 use reverse scoring: 5 = *Disagree*, 4 = *Somewhat disagree*, 3 = *Neither agree nor disagree*, 2 = *Somewhat agree*, 1 = *Completely agree*

### Factors affecting doctors’ choice of PEG for enteral nutrition

Table 5 shows the mainly reasons that affected clinicians to make PEG decision. All the items gained a high score which ranged from 3.54–3.91. For the statement about PEG is an invasive procedure and patients’ acceptance is low, the level of agreement was very high, 3.91. There were no significant differences in responses between the departments. Participants were also encouraged to supplement other reasons that had affected them, and we found that clinicians in neurology had difficulty predicting the persistence of dysphagia in stroke patients. They choose NG tube for patients’ nutritional support because dysphagia may recover within 7 days and PEG is not necessary.

**Table 5:** Reasons that influence clinicians to make PEG decision (n = 284)

For patients who need long-term nutritional support, the reasons that affect your choice of PEG are:	Level of agreement Mean ± SD (scale 1–5)
The operation is inconvenient and time-consuming, and requires the cooperation of an endoscopic physician	3.80 ± 0.87
PEG is an invasive procedure and patient acceptance is low	3.91 ± 0.72
NGT/ NJT can sustain the basic needs of patients and PEG tube feeding is not required	3.54 ± 0.85
PEG is not convenient for patients to self-care after discharge	3.58 ± 0.92

[1 = *Disagree*, 2 = *Somewhat disagree*, 3 = *Neither agree nor disagree*, 4 = *Somewhat agree*, 5 = *Completely agree*]

## Discussion

We found that NG tube feeding was still the most commonly used method for nutritional support because it’s non-invasive, convenient, fast, and affordable. In addition, the frequency of PEG use varied among different departments. Radiotherapy departments prefer to use PEG compared to other departments, possibly because nasal feeding is no longer possible for patients with advanced digestive tract or maxillofacial tumors [14]. Tertiary class A hospitals are better equipped, more technologically advanced, and have a stricter environment, making it more likely to choose PEG for enteral nutrition than class B hospitals. Additionally, clinicians working in high-grade hospitals have more opportunities to participate in some training programs. Hospitals’ attitudes may influence clinicians' decision-making processes and limit their choices.

We then surveyed doctors’ knowledge of PEG and compared them with their corresponding demographics. The result indicates that doctors' knowledge of PEG is very limited, which may lead to inappropriate decision-making. Our study found that doctors’ better understanding of the benefits of PEG comes with longer work experience, higher educational background and professional level, and the grade of hospital also contributes. Additionally, we categorized departments such as paediatrics, rheumatology and endocrinology, where enteral nutrition is not frequently applied, as “other”, and the results showed that doctors in these departments were less knowledgeable about PEG than in other departments.

There were also some contradictions. Clinicians acquired high scores in the part of indications and contraindications of PEG while when the question talked about their clinical practice, we got completely contradictory answers. The participants in this survey had a high level of agreement about the item that stated “PEG is unnecessary if NGT can sustain patients’ needs though better outcome can predict with PEG in those patients”. This demonstrates the gap between theory and practice in Chinese clinical environment, and emphasized the importance of PEG-training in hospitals. Actually, patients with NG tube feeding cannot meet their daily calorie requirements and an inadequate intake may have led to persistence of malnutrition in these long-term feeding patients [12]. Complications of long-term NG tube feeding may explain the worse prognosis of these patients. Tube dislodgement and clogging lead to frequent re-insertion which may induce nasopharyngeal area trauma and insufficient energy intake [15–17]. Some studies have confirmed that prolonged feeding with NGT implied a higher risk of aspiration and pneumonia than PEG especially in those patients with stroke-associated dysphagia [1, 18, 19].

PEG feeding has many benefits for patients with long-term enteral nutrition, so why are doctors reluctant to choose it? What has influenced their decision-making? We listed four reasons that have been frequently mentioned in previous studies [20–22], all of which had a high level of consensus in our study. The reasons listed can be divided into three domains: multidisciplinary communication is insufficient [23, 24], patients and relatives’ traditional mindset [25] and deficiencies in knowledge about PEG tube feeding among clinicians [26].

Insufficient knowledge about PEG and stereotype of the nutritional supporting methods among our clinicians further affect PEG using. Educational programs and training courses related to PEG feeding are necessary for improving the lack of knowledge and skills of HCPs. For patients who need but are hesitant to use PEG for long-term nutritional support, doctors with adequate knowledge and familiarity about PEG are more persuasive and trustworthy. Furthermore, influenced by cultural contexts for millennia, Chinese doctors prefer “conservative” and “traditional” treatments. The development of clear guidelines and enforcement standards by the hospital may help to gradually change this situation. Hospital should formulate its own Enteral Nutritional Protocol according to the national guidelines to assist doctors in making decisions.

Additionally, we should notice that the decision-making process is not only involved doctors but also patients and their relatives. In our study, the resistance of patients and their families was a main obstacle for clinicians to make PEG decisions, with a score of 3.91. Similarly, this phenomenon also has been reported in several studies [11, 21, 27].

In Asian, many countries have their own social values and are profoundly influenced by their culture and social norms which made combined effect on decision-making regarding treatment and health care options. Filial piety influences Chinese families' perceptions of the body, but providing long-term nutritional needs through safer PEG feeding methods is paradoxically rejected because of concerns about loss of body integrity [11].

On the other hand, due to the large population in China, medical resources are relatively scarce and doctor-patient relationship is tense and full of contradictions. In order to reduce unnecessary disputes, the final decision-maker of patients’ treatment plan in China are their relatives instead of doctors, unlike some European countries such as England and Whales that best decisions were made by physicians [28]. The power dynamics between doctors and patients is realigned by limiting doctors’ power over patients’ interests and encouraging patients to make autonomous clinical decisions for their own health [29], which also absolves doctors of some responsibility. However, the information acquired by patients and their families were limited and communication was also not enough. Hierarchical diagnosis and treatment system have not been fully established in China, numerous patients seek medical care from large hospitals in urban areas without a referral from a primary care institution, which puts doctors working in big hospitals under heavy work pressure. They do not have much time and energy to communicate treatment plans in detail with individual patients. [30]. The asymmetry of medical information between physicians and patients is a crucial reason for patients’ distrust of clinicians and has already affected the medical decision-making process. As a result, doctors give a suggestion and decisions are often made by patients and relatives based on inadequate information and limited medical knowledge [28, 31, 32]. Unsurprisingly, patients and their families refused using PEG feeding for long-term nutritional support because of traditional mindset and precarious trust induced by inadequate patients-physicians interaction.

Multidisciplinary cooperation between different departments should also be valued. Most of the participants thought the PEG inserted process was cumbersome and endoscopic assistance, so they were unwilling to use. Simplify the procedure of PEG placement and build a multidisciplinary nutrition team to help with decision-making is very imperative. Comparing with other departments, we found gastroenterology had a lower level of agreement about the statement that PEG insertion process is time-consuming maybe since that was their area of expertise.

Furthermore, for neurology physicians, a predictive tool which can help them estimate the duration of dysphagia is important to assist with artificial feeding decisions. It will support decision making for NGT or PEG insertion after ischemic stroke and is a step towards personalized medicine ^33^.

## Conclusions

Our present study surveyed the attitudes of clinicians from different departments toward PEG feeding. We found three objective reasons that may influence their PEG decision-making process: (1) HCPs insufficient knowledge of PEG feeding; (2) resistance from patients and families; (3) poor cooperation among HCPs and departments. There are also some subjective factors which need further research. Actually, in addition to the traditional beliefs of Chinese physicians, the poor foundation of trust between clinicians and patients also influences the medical decision-making process and leads physicians to prefer more conservative treatment to avoid disputes rather than better ones. We speculate the vital factor for this may lie in the inevitable form of China medical system caused by the large population, such as hospitals’ inappropriate internal incentives and the heavy workload of HCPs.

## Limitations and future recommendation

This study has only explored the opinion of clinicians, while patients, relatives and societal factors undoubtedly play a role in the acceptance and use of PEG feeding. Future research should therefore explore the factors influencing decisions on the route of enteral feeding from a non-HCP perspective. Additionally, the influence of the cultural barrier and medical system limitations as well as the public health literacy to PEG feeding in China needs further study and exploration.

## Supplementary Information

**Additional file 1. **Questionnaire

## Data Availability

The datasets used and/or analysed during the current study are available from the corresponding author on reasonable request.
