# Supplementary material for: Clinician's attitude to enteral nutrition with percutaneous endoscopic gastrostomy: a survey in China
Source: J Health Popul Nutr. 2021 Sep 26;40:42. doi: 10.1186/s41043-021-00264-9 (PMC8474729; doi:10.1186/s41043-021-00264-9)
Supplement: Supplementary file 1 — Additional file 1. Questionnaire [file 41043_2021_264_MOESM1_ESM.docx]

***Supplementary Material***

**Supplementary Table 1.**

**Questionnaire**

***The opening of the survey:***

*Dear doctors, this questionnaire has been developed by the first affiliated hospital of Soochow University to assess clinicians' views on the use of percutaneous endoscopic gastrostomy (PEG) for nutritional support. It would be appreciated if you took time to answer the questions of this questionnaire. Please kindly note that all data will remain confidential and that you could leave the study at any stage. Prior to taking part in the study, please click on the "I agree" box to indicate your agreement to the study.*

**Participation in the survey: □ I agree □ I disagree**

**1.Please indicate your current area of specialty：**

□ICU □ Neurology □ Neurosurgery □Rehabilitation Dept. □General surgery □ Gastroenterology □ Oncology □ Radiotherapy Dept.

□Other

**2.Ages：**

□20-30 years □30-40years □40-50years □＞50years

**3.Educational background：**

□College □Bachelor □Master □Doctor

**4.Please indicate the degree of your hospital:**

□Tertiary class A hospital □ Tertiary class B hospital □ Secondary class A hospital

□Secondary class B hospital □Other：

**5. Please indicate your working life:**

□＜3 years □ 3-5 years □5-10 years □ ＞10 years

**6. Please indicate your current status：**

□Resident □Attending □Deputy Chief □Chief

**7. Based on your actual situation, how often do you choose the following enteral nutrition methods?**

[*Never, Almost never, Sometimes, Almost always, Always*]

Nasogastric tube (NGT)

Nasal jejunal tube (NJT)

Percutaneous endoscopic gastrostomy (PEG)

Percutaneous endoscopic jejunostomy (PEJ)

**8. How much do you know about percutaneous endoscopic gastrostomy (PEG)?**

[*know nothing, do not know much, know some of it, know well, very clear*]

**9. Based on your actual situation, would you recommend PEG for the following patients who cannot be taken orally and may need long-term nutritional support?**

[*Never, Almost never, Sometimes, Almost always, Always*]

Patients with advanced dementia

Patients in terminal or palliative care

Patients with multiple sclerosis or amyotrophic lateral sclerosis

Patients with maxillofacial tumor

For stroke patients, I will place a nasogastric tube / nasal jejunum tube after the diagnosis of oropharyngeal swallowing disorder. If the swallowing disorder persists for 2 weeks, I will ask the patient's wishes, consider and recommend using PEG.

Patients with intolerance of nasogastric tube and nasal jejunal tube with complications such as reflux, gastroparesis, and gastric retention.

**10. Do you agree with the following statement about PEG?**

[*Disagree, Somewhat disagree, Neither agree nor disagree, Somewhat agree, Completely agree*]

PEG can only be used in accordance with the patient's condition and his own wishes.

Although patients with PEG feeding may have a better prognosis, but NGT/ NJT can already sustain the basic needs of patients, there is no need for PEG.

I will recommend the type of enteral nutrition to be used according to the patient's condition and guidelines. If it is contrary to the patient's wishes, I will communicate with them to explain the reasons for making decisions. In case of conflict, the patient's wishes shall prevail.

If the PEG placement process can be simplified, it will be more conducive for me to make PEG decisions.

Establishing a multi-disciplinary nutrition decision-making team will help me better choose nutrition support methods.

**11****. For patients who need long-term nutritional support, the reasons that affect your choice of PEG are:**

[*Disagree, Somewhat disagree, Neither agree nor disagree, Somewhat agree, Completely agree*]

The operation is inconvenient and time-consuming, and requires the cooperation of an endoscopic physician.

PEG is an invasive procedure and patient acceptance is low.

Nasogastric tube / Nasal jejunum tube can sustain the basic needs of patients, and PEG tube feeding is not required.

PEG is not convenient for patients to self-care after discharge.

Others: (Supplement other reasons that may affect your PEG decision)
